# A Case of Primary Malignant Peritoneal Mesothelioma

**DOI:** 10.7759/cureus.93864

**Published:** 2025-10-05

**Authors:** Liz Betancourt, Jennifer Wallace, Eli Sugarman, Bryant Estevez, Nick La Roz, Evert Castillo, Rolando Torres, Damian Casadesus

**Affiliations:** 1 Internal Medicine, Ross University School of Medicine, Miami, USA; 2 Internal Medicine, St. George's University School of Medicine, Miami, USA; 3 Psychiatry, Ross University School of Medicine, Miami, USA; 4 Internal Medicine, American University of the Caribbean, Miami, USA; 5 Internal Medicine, Jackson Memorial Hospital, Miami, USA

**Keywords:** asbestos exposure, cancer, mesothelioma, peritoneal, peritoneal mesothelioma

## Abstract

Primary malignant mesothelioma (MPM) is a rare malignancy that arises from the epithelial cells that line visceral cavities. The primary pleural presentation is known as the most commonly seen presentation and is best recognized clinically. Less commonly, mesothelioma can arise from epithelial cells lining other visceral cavities, including the peritoneum and pericardium. Primary peritoneal presentations are often difficult to diagnose due to vague symptoms such as abdominal distention, anorexia, and diffuse pain. These symptoms overlap with those of many more common gastrointestinal conditions, often leading to delayed diagnosis.

We report a male in his 40s with a past medical history of hypertension and diabetes mellitus type 2 who presented to the emergency room with non-specific abdominal complaints. He had a history of longstanding work on demolition sites. Treatment was initiated following studies that tested positive for *H. pylori* infection. A CT scan of the abdomen revealed nodular fat stranding of the omentum, with several confluent peritoneal nodules and small-volume ascites. An omental biopsy showed tumor cells positive for markers indicative of epithelioid-type malignant mesothelioma, with the peritoneum identified as the primary site.

A key piece of this patient's case was long-standing exposure to demolition sites and asbestos. Asbestos exposure is a well-documented risk factor for the development of mesothelioma. This history was obtained but did not aid in guiding diagnostic practices due to overlapping symptomatology. The discovery of an *H. pylori* infection may have delayed the diagnosis of primary malignant peritoneal mesothelioma (PMPM) and its appropriate treatment, as *H. pylori* is a much more common condition.

## Introduction

We report a case of epithelioid primary malignant peritoneal mesothelioma (PMPM) in a Hispanic male with long-term occupational asbestos exposure, with the aim of emphasizing the diagnostic delay due to confounding H. pylori gastritis and Peritoneal Cancer Index (PCI) staging and prognosis. We emphasize that a detailed history, particularly occupational history, is a critical component of clinical assessment, alongside imaging and symptomatology.

Malignant mesothelioma (MPM) is an uncommon, aggressive tumor arising from mesothelial cells lining the pleura, peritoneum, pericardium, and tunica vaginalis. The pleural form accounts for most cases, while PMPM represents 8%-10% of cases in the United States, with an annual incidence of approximately 0.5-3 per million [1].

The pathogenesis is strongly linked to asbestos fiber exposure, with additional associations including simian virus 40 (SV40) contamination, germline breast cancer gene 1-associated-protein-1 (BAP1) mutations, prior abdominal radiation, and chronic peritoneal inflammation [2]. The latency period between asbestos exposure and mesothelioma development often exceeds two decades. Compared with the pleural variant, PMPM frequently presents with non-specific symptoms. These include abdominal distension, discomfort, ascites, early satiety, and weight loss. The wide spectrum of symptoms mimics various gastrointestinal, hepatic, or gynecologic conditions. These factors may have led to diagnostic delays that worsened prognosis [3].

Histologically, the epithelioid subtype is most common in PMPM and confers a relatively favorable prognosis, with median survival approaching 51 months, versus biphasic (3 months) and sarcomatoid (6 months) variants. Treatment typically involves cytoreductive surgery (CRS) with hyperthermic intraperitoneal chemotherapy (HIPEC) when feasible, or systemic chemotherapy for unresectable disease [4]. Staging PMPM is challenging due to its diffuse spread within the peritoneum. PCI is a validated tool for quantifying tumor burden across 13 abdominopelvic regions, with higher scores predicting worse outcomes and lower likelihood of complete cytoreduction [5]. 

Ethnicity is rarely discussed in PMPM literature. While Hispanic patients represent 13.5% of cases in U.S. registry data, outcome disparities remain poorly studied [6].

## Case presentation

We present a case of a Hispanic male in his 40s with a past medical history of hypertension and type 2 diabetes mellitus, who presented to the emergency department with non-specific complaints of worsening abdominal discomfort and distension for the past six weeks. During this time, he first noticed abdominal distension accompanied by new-onset nausea, which he had not previously experienced. He also reported constipation and early satiety during the same period. He denied any history of alcohol or drug use. Notably, he endorsed long-standing occupational exposure to debris from the demolition of old buildings, without the use of a respirator. Abnormal laboratory findings are summarized in Table 1. All other results from the CBC, CMP, HIV, and hepatitis panels were within normal limits. The abnormal findings suggested possible systemic inflammatory processes, and elevated tumor markers raised suspicion of an underlying malignancy.

**Table 1:** Relevant laboratory findings

Test	Patient finding	Reference range
Albumin	3.8 g/dL (low-normal)	3.5-5.0 g/dL
ESR	28 mm/hr (elevated)	0-15 mm/hr
CRP	8.6 mg/L (elevated)	<3 mg/L
CA-125	98 U/mL (elevated)	<35 U/mL

On physical exam, the patient was alert and oriented but in evident discomfort. Mucus membranes were adequately perfused, and he appeared well hydrated; the sclera was anicteric. Lungs were clear to auscultation bilaterally, and the patient was normotensive and normocardic. His abdomen was protuberant with rigid and irregular contours. Bowel sounds were also present, but low in volume. The abdomen was diffusely tender to superficial and deep palpation; pain was worst in the left lower quadrant. A nodular pattern was evident on palpation. 

The patient underwent an upper endoscopy, which revealed only a small necrotic ulcer in the gastric antrum and transitional mucosa, consistent with active H. pylori gastritis. This was treated with appropriate antibiotics. A colonoscopy identified an 8 mm semi-sessile adenomatous polyp in the sigmoid colon, which was negative for colonic malignancy. Overall, there was no evidence of metastatic cancer throughout the gastrointestinal tract.

**Figure 1:**
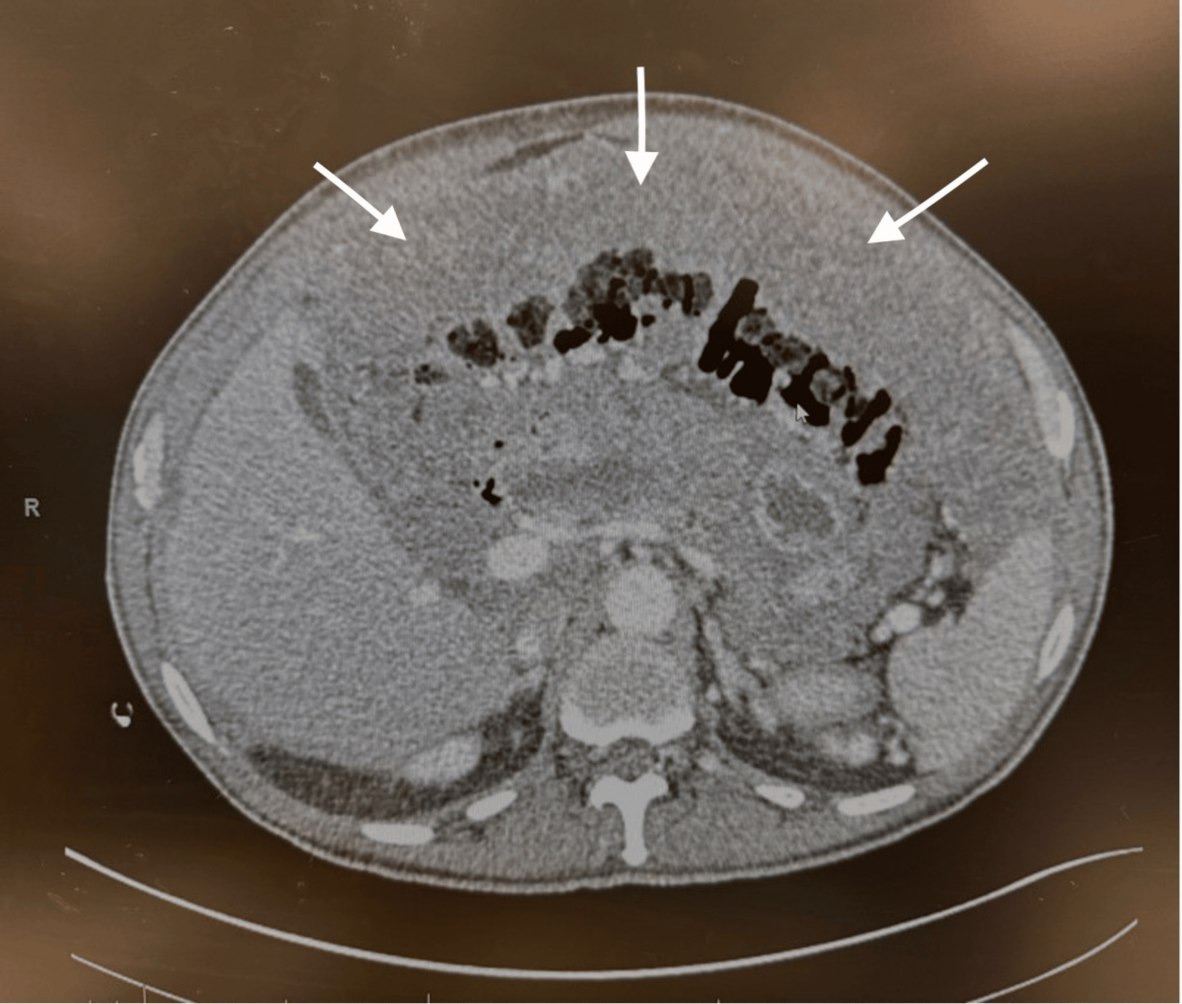
Axial CT scan shows arrows pointing to nodular implants in the abdominal peritoneum, measuring 1.4 cm and 1.8 cm

Initial abdominal computed tomography demonstrated nodular fat stranding of the omentum, with several confluent peritoneal nodules and small-volume ascites. The largest nodules were located in the left lower quadrant and within the pelvis, measuring 1.4 cm and 1.8 cm, respectively (Figure 1). No abdominopelvic lymphadenopathy was visualized. The visualized chest cavity showed no pleural nodularity, only a small calcified granuloma, which was not suspicious for malignancy. This initial imaging helped refine the differential diagnosis away from mechanical obstruction or infectious processes, as no evidence of either was observed on CT.

The patient obtained an omental biopsy that showed tumor cells positive for pan cytokeratin (AE1/AE3), calretinin, and Wilms Tumor 1 gene (WT-1). Findings support the diagnosis of epithelioid-type malignant mesothelioma with the peritoneum as the primary site. The patient also underwent two paracenteses, with ascitic fluid testing positive for carcinoma. Positron emission tomography demonstrated multiple right diaphragmatic lymph nodes with increased uptake; standardized uptake value of 3.0 (SUV >2.0, suggestive of malignancy). There were no pulmonary nodules. Omental and peritoneal uptakes significantly increased with SUVs ranging from 4.5 to 6.3. Findings were indicative of bi-cavitary disease centralized in the peritoneum. 

The patient was initiated on cisplatin (75 mg/m²) and pemetrexed (500 mg/m²) every three weeks for six cycles, followed by bevacizumab maintenance. After two months on maintenance therapy, repeat imaging demonstrated disease progression. Second-line treatment with ipilimumab (1 mg/kg) and nivolumab (3 mg/kg) every three weeks was initiated, based on data from the Intergroupe Francophone de Cancérologie Thoracique-1501 Malignant Pleural Mesothelioma and Nivolumab Study 2 (IFCT-1501 MAPS2) trial, which supports the use of checkpoint inhibitors in mesothelioma.

## Discussion

The case presented is of critical importance due to the unusual manifestation of mesothelioma in the peritoneum. Peritoneal mesothelioma accounts for approximately 8%-10% of all mesothelioma cases in the United States [1]. In this patient, the peritoneum was the primary site of oncogenesis, contributing to the uniqueness of the case. Peritoneal mesothelioma carries a differing clinical presentation from the more common pleural type. These missing features included the absence of dyspnea, chest pain, or appreciable pleural effusion in the patient presented [2]. Furthermore, this man presented with general malaise, constipation, and abdominal distention to the hospital. These common symptoms make the diagnosis of PMPM more challenging, as other high-incidence conditions can easily explain the presentation. Our patient’s symptoms overlapped with gastritis and colonic polyp findings, which initially diverted the work-up. This delay is common, with one series reporting a median time from symptom onset to diagnosis of approximately 122 days [3].

Variance of histology findings coupled with low clinical exposure for physicians makes the diagnosis of peritoneal mesothelioma a challenge [3]. The staging of peritoneal mesothelioma remains challenging due to its rare incidence and irregular lymphatic metastasis [2]. PCI divides the abdomen into 13 regions, assigning lesion scores (0-3) based on size, then summing to yield a score from 0-39 [5]. Scores >20 are associated with reduced survival and poorer outcomes after CRS and HIPEC. Our patient’s PCI of 18 indicated moderate tumor burden, likely contributing to the decision for systemic therapy rather than surgical cytoreduction. One other consideration could be using immune checkpoint inhibitors, which are an emerging second-line option with reported response rates of 15%-20% [7]. It remains difficult to assess how impactful early intervention is for patients with PMPP, but the literature suggests that patients with a lower PCI and shorter disease lead time experience improved outcomes after CRS and HIPEC [5]. 

Current literature on peritoneal mesothelioma overwhelmingly describes Caucasian individuals (75.2%) as the primary affected group, followed by Hispanics (13.5%), Black individuals (5.8%), and other (5.0%) as the remainder [6]. The described patient was a Hispanic individual, making this report more relevant. No data describing survival rates for specific ethnic groups could be found, highlighting the importance of documenting cases outside the Caucasian patient population. A study encompassing survivorship data based on ethnicity could prove to be a highly valuable endeavor. We hope that by describing this patient, earlier intervention can be promoted for improved outcomes.

## Conclusions

This case illustrates the significant impact that a rare and difficult-to-diagnose cancer can have on an individual. PMPM should be considered in patients presenting with unexplained ascites and peritoneal nodularity, particularly those with a history of asbestos exposure. A comprehensive occupational history, early imaging, and prompt biopsy are critical for timely diagnosis. PCI scoring aids in prognostication and treatment planning. Early recognition of PMPM enables timely interventions, which are essential for improving patient outcomes. It is well established that the earliest possible interventions lead to the best possible outcomes in patients with similar illnesses. Therefore, any advancement in early recognition is crucial to patient health. Identifying and clarifying common traits among patients diagnosed with peritoneal mesothelioma could significantly improve early detection, intervention, and overall survival.
